# Hypoglycemic and anti-depressant effects of Zuogui Jiangtang Jieyu formulation in a model of unpredictable chronic mild stress in rats with diabetes mellitus

**DOI:** 10.3892/etm.2014.1681

**Published:** 2014-04-14

**Authors:** YU-HONG WANG, LING-TAO YIN, HUI YANG, XIN-LIANG LI, KAI-GE WU

**Affiliations:** 1 Hunan Key Laboratory of Chinese Materia Medica Powder and Innovative Drugs Established by Provincial and Ministry, Changsha, Hunan 410007, P.R. China; 2 Key Laboratory of Internal Medicine of Traditional Chinese Medicine of Ministry of Education, The First Affiliated Hospital, Hunan University of Traditional Chinese Medicine, Hunan 410007, P.R. China; 3 College of Pharmacy, Hunan University of Chinese Medicine, Changsha, Hunan 410208, P.R. China; 4 Department of Traditional Chinese Medicine, Xiangya Hospital, Central South University, Changsha, Hunan 410219, P.R. China

**Keywords:** diabetes mellitus with depression, Zuogui Jiangtang Jieyu formulation

## Abstract

This study aimed to investigate the hypoglycemic, lipid-lowering and antidepressant effects of Zuogui Jiangtang Jieyu formulation (ZGJTJY) in a model of unpredictable chronic mild stress (UCMS) in rats with diabetes mellitus (DM; the UCMS-DM model). Sixty rats were randomly divided into blank control, vehicle (model plus vehicle), positive control (model plus metformin and fluoxetine), and high, medium and low dose ZGJTJY (model plus high, medium and low doses of ZGJTJY, respectively) groups. Following establishment of DM by a high-fat diet with intraperitoneal injection of streptozotocin (38 mg/kg), the depression model was established by application of UCMS for 28 days. The behavioral scores of the rats were detected in an open field test and Morris water maze test. The levels of blood glucose, glycosylated hemoglobin (HbA1c) and blood lipids were assayed. The total scores of the open field test and the space exploration times (SETs) in the Morris water maze test were significantly lower and the escape latency (EL) times in the Morris water maze test were significantly longer in the vehicle group compared with those in the blank control group. In addition, in the vehicle group, the levels of blood glucose, HbA1c, total cholesterol (TC), triglycerides (TGs) and low-density lipoprotein cholesterol (LDL-C) were significantly higher and the levels of high-density lipoprotein cholesterol (HDL-C) were significantly lower compared with those in the blank control group. The high dose of ZGJTJY decreased the locomotor activity levels in the open field test, the EL times of the model on day 4, the SETs in the Morris water maze test and the HDL-C levels, and reduced the blood glucose, HbA1c, TC, TG and LDL-C levels compared with those in the model group. Thus, ZGJTJY is a potential candidate for the prevention and treatment of the comorbidity of depression with DM.

## Introduction

Depression is a psychiatric disorder that presents as a reduction of confidence in oneself, the world and the future (1). At present, depression is a common mental disorder in the general population. Almost 20% of individuals in the Western world succumb to a depressive episode during their lifetime (2). Depression is the leading cause of disability among individuals worldwide and was one of the ten most common diseases globally in 2001 (3).

In a cross-sectional survey of a sample of 98,658 Chinese adults in 2010, 11.6% had diabetes mellitus (DM), of which 8.1% cases were newly detected (4). The world prevalence of DM among adults (20–79 years old) was 6.4% in 2010, affecting 285 million adults, and is predicted to increase to 7.7%, equating to 439 million adults, by 2030 according to theoretical calculations (5).

There has been a significant, increasing trend in the prevalence of depression among the diabetic population in recent years in Taiwan (6). Several meta-analysis studies have shown that the risks of elevated levels of depression and of incident depression are increased in individuals with type 2 DM compared with those in healthy subjects (7,8). Furthermore, in patients with DM and poor disease control, depression is an important risk factor for poor patient adherence to medications, but not lack of treatment intensification by physicians (9).

The coexistence of DM and depression is associated with significant morbidity, mortality and increased healthcare cost (10). Comorbid depression in individuals with DM causes a serious threat to quality of life (11). A study on the treatment of depression in diabetic patients has revealed that classical antidepressants, including monoamine oxidase inhibitors, induce hypoglycemia and weight gain, whereas tricyclics lead to hyperglycemia and carbohydrate cravings (12).

Therefore, there is an urgent requirement to identify an agent with greater efficacy and fewer side-effects for treating comorbid depression with DM.

In comorbid DM and depression, the majority of the evidence supports the use of fluoxetine in the control of glucose handling (13). However, selective serotonin reuptake inhibitors may cause discontinuation or withdrawal symptoms, including nausea, vomiting and diarrhea (14), which are induced by the inhibition of gastric motor activity (15). Metformin is the mainstay treatment in the control (16) and prevention (17) of DM and associated comorbidities (18).

In traditional medicinal systems, numerous herbal drugs may be combined to produce multi-herbal formulas that enhance the effects and reduce the toxicity of the individual drugs. The purpose of the present study was to demonstrate the hypoglycemic, lipid-lowering and antidepressant effects of Zuogui Jiangtang Jieyu formulation (ZGJTJY) in a model of unpredictable chronic mild stress (UCMS) in rats with DM. The experimental protocol was in accordance with guidelines of Hunan University of Traditional Chinese Medicine and the Guide for the Care and Use of Laboratory Animals (NIH publication no. 80-23, 1996) and was approved by the Institutional Animal Care and Use Committee of Hunan University of Traditional Chinese Medicine.

## Material and methods

### Traditional Chinese medicine preparation

The ZGJTJY formulation consisted of 11 herbal components: *Astragalus membranaceus* (18.0 g), *Hypericum perforatum* (St. John’s wort; 3.0 g), rhizome of *Curcuma Longa* (9.0 g), prepared *Rehmannia* root (15.0 g), *Cornus offinalis* (12.0 g), *Lycium barbarum* L. (12.0 g), *Cuscuta chinensis* seed (9.0 g), *Eucommia ulmoides* (9.0 g), *Salvia miltiorrhiza* (12.0 g), root bark of *Paeonia suffruticosa* (6.0 g) and *Achyranthes* root (9.0 g). It was provided by the pharmacy of The First Affiliated Hospital, Hunan University of Traditional Chinese Medicine (Changsha, China). The ZGJTJY was boiled, filtered and concentrated to a 2.28-g/ml liquid at 80°C in a water bath and stored at 4°C in a refrigerator. When used, it was diluted with distilled water and administered by gavage.

### Drug and materials

Metformin hydrochloride tablets were purchased from Hunan Xiangya Pharmaceutical Co., Ltd. (Changsha, China; Lot: 1303106; 0.25 g/tablet); fluoxetine hydrochloride capsules were obtained from Patheon France S.A.S (Bourgoin Jallieu, France; Lot: 0972A; 20 mg/tablet); and streptozotocin (STZ) was purchased from Sigma-Aldrich (St. Louis, MO, USA). The high-fat diet (HFD) consisted of 58% fat, 25% protein and 17% carbohydrate, as a percentage of the total kilocalories. The high-speed refrigerated centrifuge was from Sigma-Aldrich (SIGMA 3K15, Sigma Laborzentrifugen GmbH, Osterode am Harz, Germany), a microplate reader was obtained from Thermo Fisher Scientific Inc. (Waltham, MA, USA; MK3) and the open boxes were homemade.

### Animals and drug administration

Specific pathogen-free, male Sprague Dawley rats (weight, 200–220 g; license no. SCXK 2009-0004) were provided by Hunan Slac Jingda Laboratory Animal Co., Ltd. (Changsha, China), and all animals were housed with a 12-h light/dark cycle, in accordance with the National Institutes of Health Guide for the Care and Use of Laboratory Animals (1996).

The rats were randomly divided into the blank control, vehicle (model plus vehicle), positive control (model plus metformin and fluoxetine), and high, medium and low dose ZGJTJY (hZGJTJY, model plus high dose of ZGJTJY; mZGJTJY, model plus medium dose of ZGJTJY; and lZGJTJY, model plus low dose of ZGJTJY, respectively) groups, with 10 rats per group. The dosages of the drugs were as follows: Metformin, 1.8 mg/kg; fluoxetine, 10.8 mg/kg; hZGJTJY, 2.28 g/ml; mZGJTJY, 1.14 g/ml; and lZGJTJY, 0.57 g/ml. The rats in the model group received the same volume of distilled water. All rats were treated by gastric perfusion once per day.

### Model of UCMS in rats with DM

The experimental model of DM was induced with a combination of low-dose STZ and a HFD. Following the onset of the experiment, the rats were fed *ad libitum* with a HFD for two weeks and then received 35 mg/kg STZ freshly dissolved in citrate buffer (pH 4.5) intraperitoneally after fasting overnight (19). The rats with non-fasting plasma glucose levels of ≥300 mg/dl were considered diabetic and selected for further study.

The UCMS model was established according to the methods of Willner with modifications (20). The stress procedure contained a range of stressors, which consisted of: 24-h water deprivation, a 1-min tail pinch, 5-min thermal stimulation in a 45°C oven, 5-min cold swimming at 4°C, a 24-h reversed light/dark cycle, 48-h food deprivation, electric shock to the foot (10 mA current; administered every other minute and lasting 10 sec per time for 30 times), shaking (once per second; lasting for 15 min), noise (85 dB) and strange smell. During a period of 28 days, one of the stimuli was selected randomly and applied to the rats so that the rats were not able to expect the stimulus. Every stimulus used 2 or 3 times in total for each rat within 28 days.

### Open field test

An open field test was used to conduct scoring of the rats in all groups. The open-field device was made of opaque materials with a 80×80 cm square located on the bottom, which was equally divided into 25 equilateral squares. Surrounding the base there was a wall with a height of 40 cm. The rat was placed in the central square and then the number of squares the rat crossed in 5 min was measured (only the squares that the rat entered on four feet were included in the score of horizontal activity) and the number of times the rat stood on hind limbs (the score of vertical activity) was observed. Each rat was measured once for 5 min, which was scored by two observers and the average value was recorded. The sum of the horizontal and vertical activity scores was considered to be indicative of the locomotor activity (LMA).

### Morris water maze test

Spatial learning and memory were observed in the Morris water maze using procedures similar to those described previously (21). The Morris water maze consisted of a circular fiberglass pool (200 cm in diameter) filled with water (25±1°C) and made opaque with black non-toxic paint. The pool was surrounded by light blue curtains and three distal visual cues were fixed to the curtains. Four floor light sources of equal power provided uniform illumination in the pool and testing room. A charge-coupled device camera (kl-9511zh, Konlan Company, Shuozhou, China) suspended above the pool center recorded the swim paths of the animals and the video output was digitized by an EthoVision XT tracking system (Noldus Information Technology, Inc., Leesburg, VA, USA).

Four trials from each of the four quadrants were conducted once a day for five days. The video analysis system tracked, recorded and analyzed the swimming speed and the time taken to locate the platform for each animal. Each trial lasted either until the rat located the platform or for 60 sec, which was recorded as the escape latency (EL) time, and the mean EL time of the last four days as the outcome of learning. The platform was removed for a 60-sec probe trial on the final day, and the time spent swimming in the platform quadrant was recorded as the space exploration time (SET).

### Detection of plasma glucose and serum lipid levels

Following the final behavioral test, a single touch glucometer (OneTouch Ultra 2; LifeScan, High Wycombe, UK) was used to determine the glucose levels in plasma collected from the tail vein of the rats. Subsequently, the rats were anesthetized, and blood samples were collected by the abdominal aortic method in tubes containing EDTA and centrifuged at 2,500 × g for 15 min at 4°C. The serum was stored at −70°C until analysis. The serum levels of glycosylated hemoglobin (HbA1c), total cholesterol (TC), triglyceride (TG), high-density lipoprotein cholesterol (HDL-C) and low-density lipoprotein cholesterol (LDL-C) were determined using enzymatic kits (Nanjing Jiancheng, Nanjing, China). All serum samples were measured with a RT-1904C Semi-auto Chemistry Analyzer (Rayto Life and Analytical Sciences Co., Ltd., Shenzhen, China)

### Statistical analysis

All results are presented as the mean ± standard error of the mean. Variance analysis was used make comparisons among the groups. P<0.05 was considered to indicate a statistically significant difference. A test for homogeneity was used to examine the data and if the data was homogeneous, a one-way analysis of variance (ANOVA) was conducted directly on the data. Between the two groups, the least significant difference method was used to compare any differences. Otherwise, the parameters were changed first and then the test for homogeneity was used again. ANOVA was only conducted on the data which became homogeneous following the change of parameters. All statistical analyses were performed using SPSS software for Windows, version 18.0 (SPSS, Inc., Chicago, IL, USA).

## Results

### Open field test

**Figure 1:**
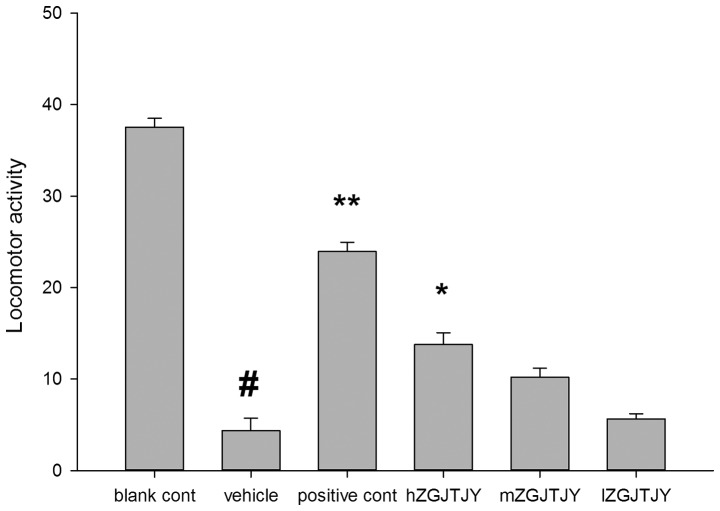
Effects of ZGJTJY on the LMA levels of rats in the open field test. Values are presented as the mean of the total scores in the open field test ± standard error of the mean. ^#^P<0.05, compared with the blank control group; ^*^P<0.05 and ^**^P<0.01, compared with the model group. Cont, control; hZGJTJY, high dose Zuogui Jiangtang Jieyu formulation; mZGJTJY, medium dose Zuogui Jiangtang Jieyu formulation; lZGJTJY, low dose Zuogui Jiangtang Jieyu formulation; LMA, locomotor activity.

As shown in Fig. 1, the total scores of the open field test were significantly lower in the vehicle group than those in the blank control group (P<0.05). Compared with those in the vehicle group, hZGJTJY increased the LMA levels of the UCMS-DM model rats (P<0.05).

### Morris water maze test

**Figure 2:**
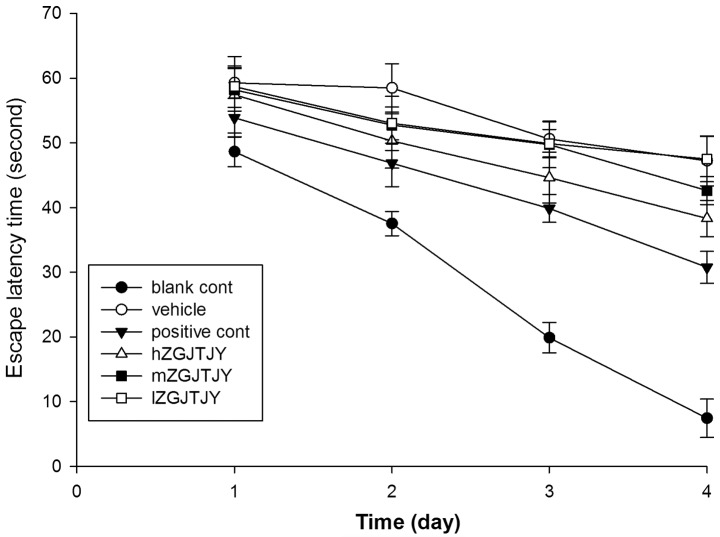
The EL times in the Morris water maze test on days 1–4 for all groups of rats. Cont, control; hZGJTJY, high dose Zuogui Jiangtang Jieyu formulation; mZGJTJY, medium dose Zuogui Jiangtang Jieyu formulation; lZGJTJY, low dose Zuogui Jiangtang Jieyu formulation; EL, escape latency.

The EL times in the Morris water maze test (Fig. 2) were significantly longer in the vehicle group than those in the blank control group on days two, three and four (P<0.05, P<0.01 and P<0.01, respectively). hZGJTJY markedly decreased the EL times of the model group on day 4 (P<0.05).

**Figure 3:**
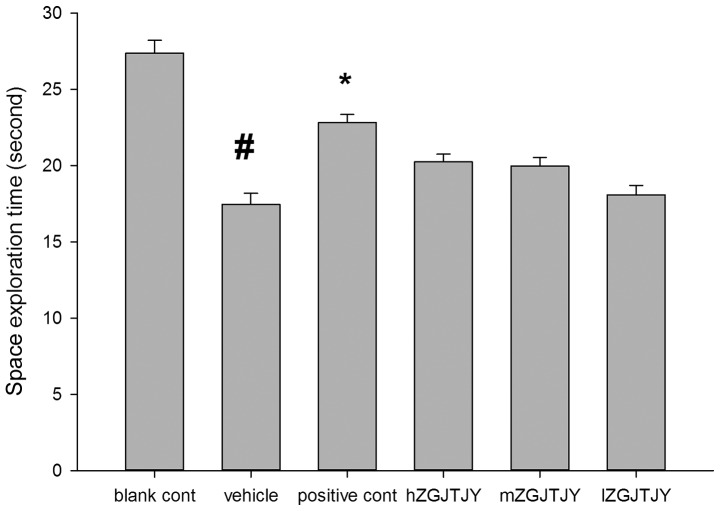
Effects of ZGJTJY on the SETs in the Morris water maze test. ^#^P<0.05, compared with the blank control group; ^*^P<0.05, compared with the model group. Cont, control; hZGJTJY, high dose Zuogui Jiangtang Jieyu formulation; mZGJTJY, medium dose Zuogui Jiangtang Jieyu formulation; lZGJTJY, low dose Zuogui Jiangtang Jieyu formulation; SET, space exploration time.

The SETs in the Morris water maze test (Fig. 3) were shorter in the vehicle group than those in the blank control group (P<0.05). Compared with those in the model group, hZGJTJY increased the SETs.

### Blood glucose and HbA1c levels

**Figure 4:**
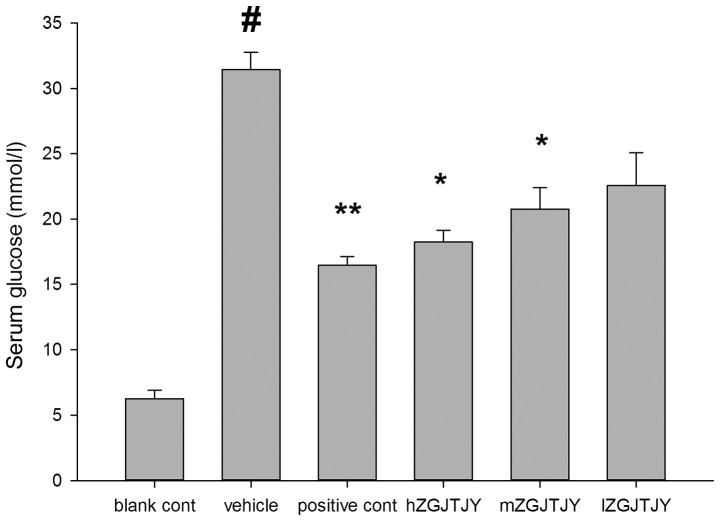
Plasma glucose levels (mmol/L) in all groups. ^#^P<0.05, compared with the blank control group; ^*^P<0.05 and ^**^P<0.01, compared with the model group Cont, control; hZGJTJY, high dose Zuogui Jiangtang Jieyu formulation; mZGJTJY, medium dose Zuogui Jiangtang Jieyu formulation; lZGJTJY, low dose Zuogui Jiangtang Jieyu formulation.

**Figure 5:**
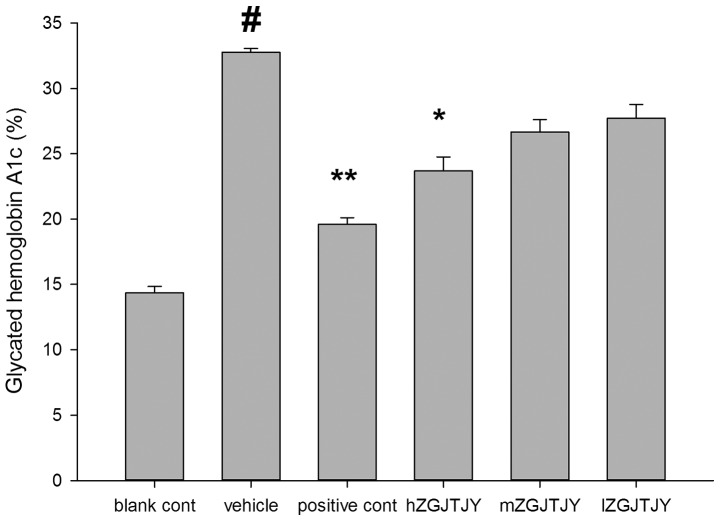
HbA1c (%) in all groups. ^#^P<0.05, compared with the blank control group; ^*^P<0.05 and ^**^P<0.01, compared with the model group. Cont, control; hZGJTJY, high dose Zuogui Jiangtang Jieyu formulation; mZGJTJY, medium dose Zuogui Jiangtang Jieyu formulation; lZGJTJY, low dose Zuogui Jiangtang Jieyu formulation; HbA1c, glycosylated hemoglobin.

The blood glucose (Fig. 4) and HbA1c (Fig. 5) levels were higher in the vehicle group than those in the blank control group (P<0.05). hZGJTJY reduced the blood glucose and HbA1c levels (P<0.05), while mZGJTJY significantly lowered the blood glucose levels of the model group (P<0.05).

### Lipid analysis

The TC, TG and LDL-C levels were significantly higher, while the HDL-C levels were significantly lower in the vehicle group than those in the blank control group. hZGJTJY reduced the TC, TG and LDL-C levels (P<0.05), and elevated the HDL-C levels (P<0.01) compared with those in the model group. mZGJTJY also significantly increased the HDL-C levels compared with those in the model group (P<0.05; Table I).

## Discussion

The comorbidity of depression with chronic physical diseases, including arthritis and DM, is well recognized in developed countries (22–24).

Comorbid depression accompanied with DM is common clinically, but the establishment of a mammalian model of it is challenging. At the initial stages of the present study, PubMed and other websites were searched and no experimental designs similar to that of the present study were found. The present study established a model of depression accompanied by DM, which comprised a combination of the two patterns of animal model. The model proposed for the first time in the present study intensifies the depression status in DM, compared with the real depression status of a patient with DM.

A previously described model of type 2 DM was induced with the combination of a HFD and low-dose STZ (19). That model simulated the human syndrome of depression with DM and was identified as suitable for testing antidiabetic agents for the treatment of type 2 DM. The chronic stress-induced depression model is an effective model for studying depression and has been widely used in basic research and drug screening for depression (25,26). The model simulates the core symptoms of depression: Loss of interest, anhedonia, and a reduction in exploratory ability and sexual behavior. Helplessness and anhedonia are the core symptoms of depression and the majority of the current models only mimic anhedonia. The currently available chronic mild stress model is possibly the most valid and widely used animal model of depression.

In summary, the present study attempted to establish a model of depression accompanied with DM for the first time, and demonstrated that a high dose of ZGJTJY increased the LMA levels in the open field test, the EL times of the model on day four of the Morris water maze test and the SETs in the Morris water maze test. In addition it increased HDL-C levels, and reduced the blood glucose, HbA1c, TC, TGs and LDL-C levels compared with those in the model group. If the action of ZGJTJY is positive in clinic, further clinical research would enhance the development of the new drug.

## Figures and Tables

**Table I:** Serum lipid content in all groups.

Group	TC	TG	HDL-C	LDL-C
Blank control	0.87±0.04	0.39±0.06	1.19±0.18	0.78±0.16
Vehicle	2.64±0.12^a^	1.73±0.08^a^	0.36±0.04^a^	1.42±0.07^a^
Positive control	1.45±0.07^b^	0.87±0.04^b^	0.89±0.08^c^	0.82±0.03^b^
hZGJTJY	1.85±0.02^b^	1.25±0.07^b^	0.74±0.06^c^	0.94±0.06^b^
mZGJTJY	2.27±0.06	1.57±0.12	0.51±0.09^b^	1.21±0.02
lZGJTJY	2.36±0.15	1.59±0.05	0.49±0.03	1.29±0.12

a P<0.05, compared with the blank control group;

b P<0.05 and

c P<0.01, compared with the model group.

TC, total cholesterol; TG, triglyceride; HDL-C, high-density lipoprotein cholesterol; LDL-C, low-density lipoprotein cholesterol; hZGJTJY, high dose Zuo-gui Jiang-tang Jie-yu formulation; mZGJTJY, medium dose Zuo-gui Jiang-tang Jie-yu formulation; lZGJTJY, low dose Zuo-gui Jiang-tang Jie-yu formulation.

## Abbreviations

ZGJTJY: Zuogui Jiangtang Jieyu formulation
UCMS: unpredictable chronic mild stress
DM: diabetes mellitus
STZ: streptozotocin
